# Combining histological grade, TILs, and the PD-1/PD-L1 pathway to identify immunogenic tumors and de-escalate radiotherapy in early breast cancer: a secondary analysis of a randomized clinical trial

**DOI:** 10.1136/jitc-2022-006618

**Published:** 2023-05-19

**Authors:** Axel Stenmark Tullberg, Martin Sjöström, Lena Tran, Emma Niméus, Fredrika Killander, Anikó Kovács, Dan Lundstedt, Erik Holmberg, Per Karlsson

**Affiliations:** 1 Department of Oncology, University of Gothenburg Institute of Clinical Sciences, Goteborg, Sweden; 2 Department of Radiation Oncology, UCSF, San Francisco, California, USA; 3 Department of Clinical Sciences Lund, Oncology/Pathology and Surgery, Lund University, Lund, Sweden; 4 Department of Surgery, Skåne University Hospital, Lund, Sweden; 5 Department of Oncology, Skåne University Hospital, Lund, Sweden; 6 Department of Clinical Pathology, Sahlgrenska University Hospital, Gothenburg, Sweden

**Keywords:** Clinical Trials, Phase III as Topic, Tumor Biomarkers, Tumor Microenvironment, Radiotherapy, Genome Instability

## Abstract

**Background:**

The implementation of immunological biomarkers for radiotherapy (RT) individualization in breast cancer requires consideration of tumor-intrinsic factors. This study aimed to investigate whether the integration of histological grade, tumor-infiltrating lymphocytes (TILs), programmed cell death protein-1 (PD-1), and programmed death ligand-1 (PD-L1) can identify tumors with aggressive characteristics that can be downgraded regarding the need for RT.

**Methods:**

The SweBCG91RT trial included 1178 patients with stage I–IIA breast cancer, randomized to breast-conserving surgery with or without adjuvant RT, and followed for a median time of 15.2 years. Immunohistochemical analyses of TILs, PD-1, and PD-L1 were performed. An activated immune response was defined as stromal TILs ≥10% and PD-1 and/or PD-L1 expression in ≥1% of lymphocytes. Tumors were categorized as high-risk or low-risk using assessments of histological grade and proliferation as measured by gene expression. The risk of ipsilateral breast tumor recurrence (IBTR) and benefit of RT were then analyzed with 10 years follow-up based on the integration of immune activation and tumor-intrinsic risk group.

**Results:**

Among high-risk tumors, an activated immune infiltrate was associated with a reduced risk of IBTR (HR 0.34, 95% CI 0.16 to 0.73, p=0.006). The incidence of IBTR in this group was 12.1% (5.6–25.0) without RT and 4.4% (1.1–16.3) with RT. In contrast, the incidence of IBTR in the high-risk group without an activated immune infiltrate was 29.6% (21.4–40.2) without RT and 12.8% (6.6–23.9) with RT. Among low-risk tumors, no evidence of a favorable prognostic effect of an activated immune infiltrate was seen (HR 2.0, 95% CI 0.87 to 4.6, p=0.100).

**Conclusions:**

Integrating histological grade and immunological biomarkers can identify tumors with aggressive characteristics but a low risk of IBTR despite a lack of RT boost and systemic therapy. Among high-risk tumors, the risk reduction of IBTR conferred by an activated immune infiltrate is comparable to treatment with RT. These findings may apply to cohorts dominated by estrogen receptor-positive tumors.

## Background

Adjuvant radiotherapy (RT) after breast-conserving surgery (BCS) significantly decreases the incidence of ipsilateral breast tumor recurrence (IBTR).^1^ However, despite standard treatment, approximately 10% of patients experience an IBTR within 10 years of diagnosis, associated with an increased risk of subsequent distant metastasis and death.^1 2^ Patients with high-risk tumors may be recommended RT boost to eliminate residual microscopic tumor foci.^3^ The most widely accepted boost indication is young age.^3^ Furthermore, other characteristics of tumor aggressivity represent additional boost indications, although the definition varies between guidelines.^3 4^ RT de-escalation has so far focused on low-risk tumors. However, recent data indicate significant prognostic heterogeneity among patients with high-risk tumors, for example, young individuals with estrogen receptor (ER)-negative tumors.^5^ This is an area where immunological biomarkers show great potential.^5^ In light of the above, we believe it is highly relevant to study the possibility of RT de-escalation in high-risk groups.

CD8+T cells are considered the primary effector cell of the antitumoral immune response^6 7^ and react to protein products of mutated tumor genes (ie, neoantigens). T cells are regulated by the programmed cell death protein-1 (PD-1)/programmed death ligand-1 (PD-L1) pathway and other immune checkpoints.^8 9^ Despite its inherent inhibitory effect on CD8+T cells, an active PD-1/PD-L1 pathway may correlate with an activated immune response and an improved prognosis among aggressive subtypes.^10^ Assessments of the PD-1/PD-L1 axis provide independent information in addition to tumor-infiltrating lymphocytes (TILs),^11^ but it is unknown if this can be used to improve RT individualization. We have previously shown that high stromal TILs may be associated with a reduced risk of IBTR and decreased RT benefits.^12^

Histological grade has long been an important prognostic factor in breast cancer and primarily measures proliferation and dedifferentiation.^13^ In a previous study, we found that a signature correlating strongly with histological grade could predict the prognostic effect of an activated immune infiltrate^14^—a characteristic we will henceforth refer to as immune responsiveness. Histological grade may thus represent tumor-intrinsic qualities that predict the biological implications of a local immune infiltrate. However, many tumors are classified as grade II, which does not provide useful clinical information.^15^ Previous studies indicate that subtype, in part, can determine immune responsiveness.^6^ Subtype correlates with proliferation, whose biological relevance is illustrated by the fact that it may explain most of the performance of prognostic breast cancer signatures.^16 17^ Because the luminal B subtype exhibits significant heterogeneity regarding proliferation,^18^ we do not believe that subtype alone is the optimal method to estimate immune responsiveness. This is supported by recent data indicating immunotherapy responsiveness among a subset of luminal B tumors.^19^ For this reason, we chose to use histological grade as a hypothesized marker of immune responsiveness in this study.

This study aimed to investigate whether an integrated analysis of TILs, the PD-1/PD-L1 signaling pathway, and histological grade can identify immune-responsive tumors from a cohort dominated by luminal tumors and inform RT de-escalation. These biomarkers are already being evaluated in clinical practice, and an increased understanding of their interaction for determining RT benefit and immune responsiveness may improve the treatment of patients with breast cancer. Using our previously developed gene expression signature predicting immune responsiveness,^14^ we also attempted to stratify grade II tumors into high-risk and low-risk groups with hypothesized different benefits of a local immune infiltrate. We hypothesized that high-risk tumors with an activated immune response could be downgraded in terms of locoregional treatment.

## Methods

### Study population

**Figure 1:**
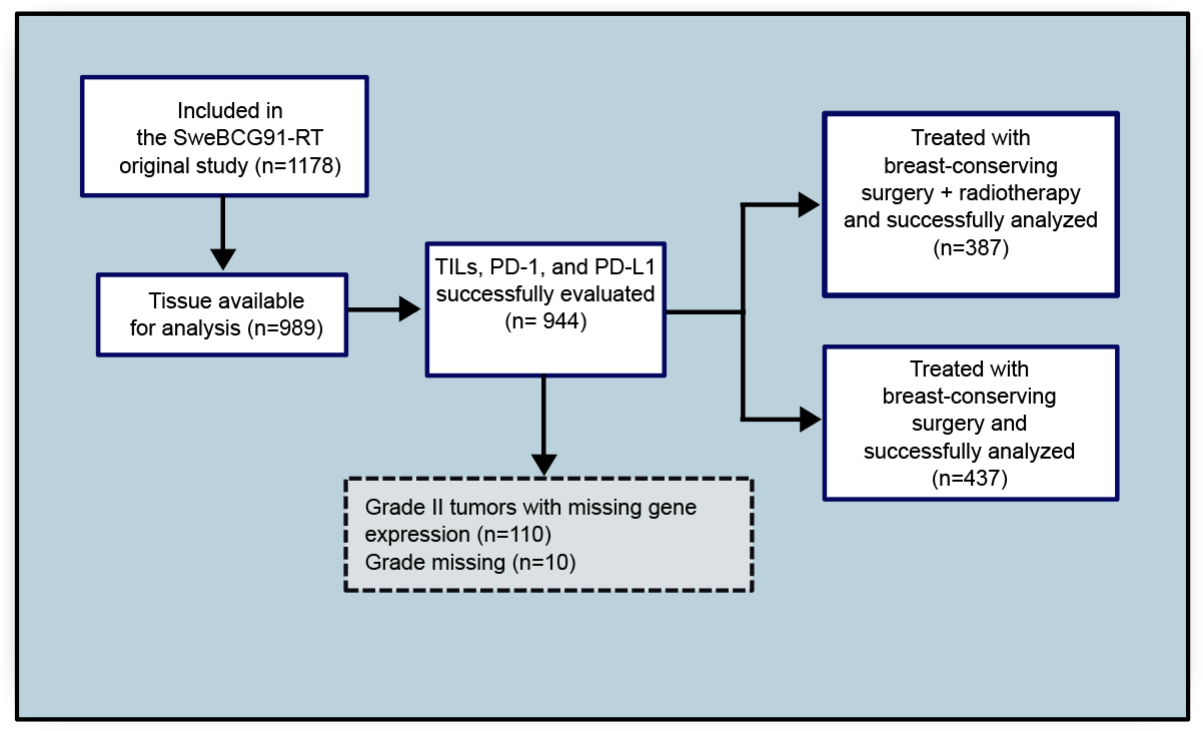
Consort diagram of included patients. Tumor blocks from patients included in the original SweBCG91RT trial were recollected. TILs and histological grade were scored on whole tissue sections and PD-1/PD-L1 were scored on TMAs. PD-1, programmed cell death protein-1; PD-L1, programmed death ligand-1; RT, radiotherapy; TILs, tumor-infiltrating lymphocytes; TMA, tissue microarray.

Patients from the SweBCG91RT trial were analyzed.^20 21^ In summary, 1178 patients with lymph node-negative (N0) stage I or IIA breast cancer were randomly assigned between 1991 and 1997 to BCS with or without whole-breast RT and followed for a median time of 15.2 years(online supplemental file 3) (figure 1). No patient had a positive surgical margin. Systemic adjuvant therapy was given per regional guidelines at the time. In total, 7% of patients received endocrine treatment, 1% received chemotherapy, and 0.4% received both endocrine therapy and chemotherapy. Tumor blocks were recollected and tumor subtyping was performed according to the St Gallen International Breast Cancer Conference (2013) Expert Panel on tissue microarray (TMA) slides as described previously.^22^ In short, tumors were classified as luminal A–like (ER-positive, progesterone receptor (PgR)-positive, human epidermal growth factor receptor 2 (HER2)-negative, and Ki-67 low), luminal B–like (ER-positive, PgR-negative or Ki-67 high, and HER2-negative), HER2-positive (HER2-positive, any ER and PgR status, any Ki-67) and triple-negative (ER-negative, PgR-negative, HER2-negative, and any Ki-67). Analyses were performed on treatment-naïve formalin-fixed paraffin-embedded (FFPE) tumor samples. Invasive carcinoma was histologically confirmed by a board-certified pathologist. Included patients did not differ from excluded patients except for histological grade and tumor size. Excluded patients had slightly smaller tumors of a lower histological grade (online supplemental table S1).

10.1136/jitc-2022-006618.supp3 Supplementary data

10.1136/jitc-2022-006618.supp1 Supplementary data

The original trial and follow-up study were conducted per the Declaration of Helsinki. Oral informed consent was obtained from all patients before performing human investigations for the original trial and this follow-up study, and was determined appropriate and approved by the Ethical Review Board.

### Data sharing

Gene expression data has been deposited in the Gene Expression Omnibus under accession number GSE119295. Due to regulations of the ethical review board and laws related to patient privacy, all clinical information has not been made publicly available.

### Immunohistochemistry evaluations

**Figure 2:**
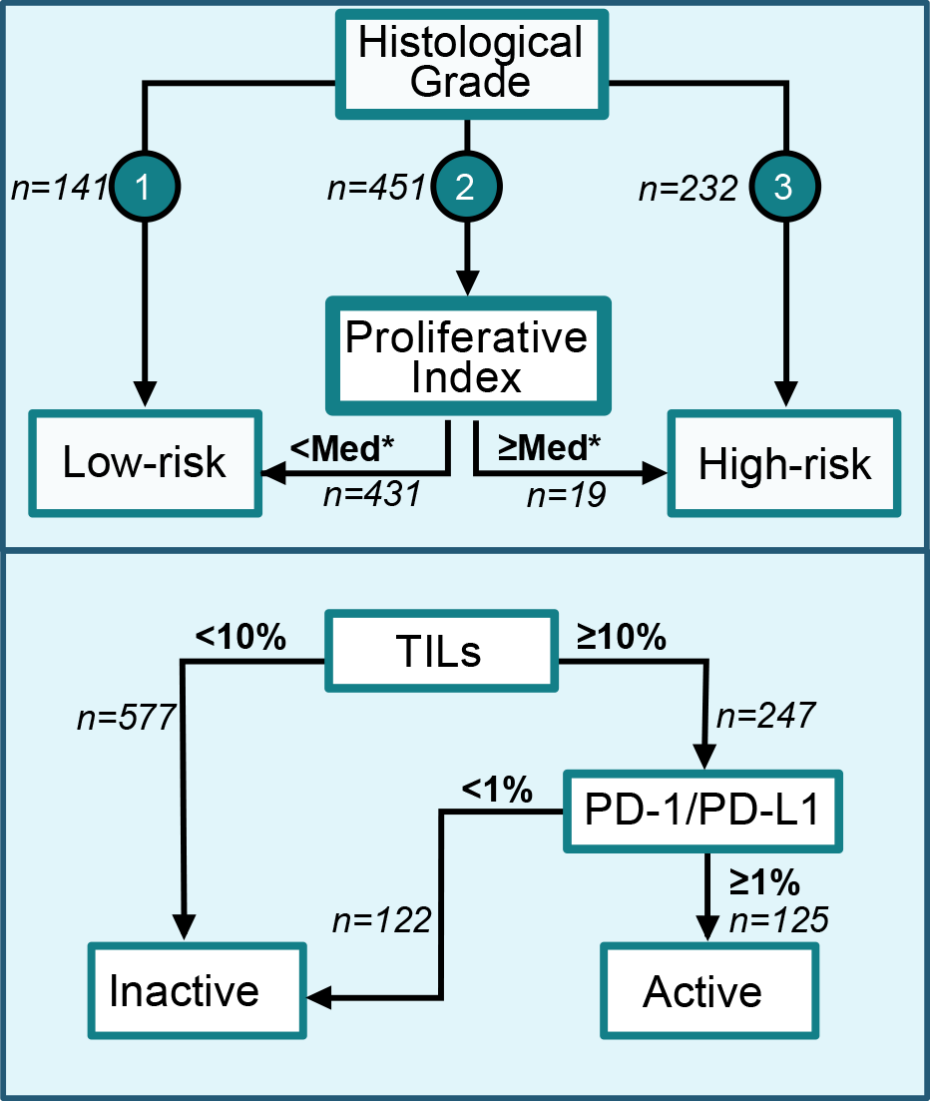
Flow charts for the classification of tumors into low-risk and high-risk tumor-intrinsic groups as well as of immune infiltrates as activated or inactivated/absent. *The median score of grade III tumors was used as the cut-off to classify grade II tumors as low-risk or high-risk. PD-1, programmed cell death protein-1; PD-L1, programmed death ligand-1; TILs, tumor-infiltrating lymphocytes.

Stromal TILs were evaluated on whole tissue H&E-stained sections as described previously.^12^ In short, TILs were evaluated as semicontinuous values (0%, 1–9%, 10–49%, 50–74%, 75–100%) by two board-certified pathologists, who were blinded to the outcome, until consensus was reached.^12^ Evaluations of PD-1 and PD-L1 were performed on TMAs by two board-certified pathologists using the Cell Marque (NAT105) and Ventana (SP142) antibodies. Two cores per marker were evaluated, and the highest value per marker was chosen, given that TMA evaluations of immune checkpoint proteins tend to underestimate the degree of positive staining.^23^ Staining of ≥1% of lymphocytes was defined as positive, as this is the cut-off used in clinical practice to determine PD-L1 positivity^24^ (an image of positive staining can be found in the online supplemental file 1). Staining protocols are included in the online supplemental file 1. We defined an activated immune infiltrate as TILs ≥10% and positive staining for at least one of PD-1 or PD-L1 (figure 2). We based this on previous literature indicating that TILs and immune checkpoint molecule expression provide independent information, complementing each other.^25^ Consequently, combining TILs with checkpoint molecule expression measurements may allow for identifying the most immunogenic tumors compared with either marker alone.^26^

### Tumor-intrinsic risk group assessment

We then divided patients into low-risk and high-risk groups depending on histological grade and the previously developed Proliferative Index signature.^14^ Histological grade I was classified as low-risk and grade III as high-risk. In our previous study, Proliferative Index demonstrated a strong correlation with histological grade and proliferation, and could predict the immune responsiveness of tumors. We hypothesized that grade II tumors are heterogeneous and can be reclassified into high-risk or low-risk as previously suggested.^27^ Most tumors of the SweBCG91RT cohort were previously classified as grade II. Since the literature indicates that an immune infiltrate’s prognostic effect in low-risk, ER-dominated, cohorts is either absent or unfavorable, we hypothesized that the majority of grade II tumors should be classified as low-risk and not immune-responsive.^28–30^ This hypothesis was further supported by the fact that the Proliferative Index of grade II tumors resembled grade I tumors more than grade III tumors (online supplemental figure S1). We, therefore, hypothesized that grade II tumors were more similar to grade I tumors regarding the biological implications of an immune infiltrate. To accurately reclassify grade II tumors based on their hypothesized immune responsiveness, we set the cut-off for high-risk grade II tumors at the median Proliferative Index of grade III tumors. The remainder of grade II tumors were classified as low-risk (figure 2). The high cut-off was further motivated by the fact that we did not want to dilute the hypothesized effect size of the high-risk group.

### Statistical methods

Time to IBTR as the first event within 10 years from diagnosis was used as the primary endpoint. The aims were to analyze the interaction between an activated immune response and tumor-intrinsic risk group (high-risk or low-risk) on the risk of IBTR and its implications for the benefit from RT. A likelihood-ratio test between regression models with and without an interaction term was used to test the interaction effect. A p value<0.05 was considered significant. P values reported for other analyses, which were not part of the main hypothesis, were not adjusted for multiple hypothesis testing and should be interpreted with caution. Hazard ratios (HRs) with 95% confidence intervals (CIs) presented in tables and the results section were calculated with cause-specific Cox proportional hazards regression to reflect the biological effect of an activated immune infiltrate depending on tumor-intrinsic risk groups in the presence of competing risks. Other recurrences and deaths were considered competing risks for IBTR. Cumulative incidences were used to describe 10-year IBTR rates. Figures of cumulative incidences were created according to the method of Fine and Gray^31^ and based on the Cox models of subhazards, producing subdistribution HRs. P values for differences in cumulative incidences between compared groups were denoted as *P_CIF_* in the plots. Age, tumor size, ER status, and RT were tested in univariable analysis and, if significant, included in multivariable analysis.

The proportional hazards assumption was checked using the Schoenfeld residuals. It was violated for histological grade and RT. Therefore, estimates for these variables should be regarded as the mean effect over the 10-year follow-up period. Due to the violation of the proportional hazards assumption, we also included analyses with a follow-up time of 5 years in the supplement (online supplemental tables S2–S4). The results of these analyses were similar to those presented in the main manuscript, and the proportional hazards assumption was not violated.

Stata V.17.0 was used for analysis (StataCorp. 2017, Stata: Release 17, Statistical Software, StataCorp).

## Results

### Demographics

In total, 148 (15.4%) tumors were classified as grade I, 573 (59.8%) as grade II, and 237 (24.7%) as grade III. We calculated the previously developed signature, Proliferative Index, and centered and standardized the scores to have a mean of 0 and an SD of 1. We then used the Proliferative Index to classify grade II tumors as high-risk or low-risk (figure 2). Grade I tumors had a median Proliferative Index of −0.70, grade II tumors −0.43, and grade III tumors 1.03 (online supplemental figure S1). A total of 19 (3.3%) of the 573 grade II tumors had a Proliferative Index equal to or higher than the median of grade III tumors and were classified as high-risk.

In total, 139 (55.4%) of high-risk tumors had high TILs (≥10%), 62 (24.7%) had a high PD-1 expression (≥1%), and 101 (40.2%) had a high PD-L1 expression (≥1%) (table 1). A total of 96 (38.2%) tumors were classified as having an activated immune response (TILs ≥10% and PD-1 and/or PD-L1 ≥1%). A total of 75 (36.1%) high-risk tumors were ER negative, 232 (92.4%) tumors were of grade III, and 19 (7.6%) were of grade II (table 1, online supplemental table S5). Tumors with TILs ≥10% and PD-1/PD-L1 expression ≥1% generally had higher TILs than tumors with TILs ≥10% but without PD-1/PD-L1 expression (online supplemental table S6).

**Table 1:** Demographics of included patients

Variables	Low-risk group			High-risk group		
	No RT	RT	Total	No RT	RT	Total
TILs						
Low	240 (81.4%)	225 (80.9%)	465 (81.2%)	63 (44.4%)	49 (45.0%)	112 (44.6%)
High	55 (18.6%)	53 (19.1%)	108 (18.8%)	79 (55.6%)	60 (55.0%)	139 (55.4%)
PD-1						
Low	267 (90.5%)	258 (92.8%)	525 (91.6%)	109 (76.8%)	80 (73.4%)	189 (75.3%)
High	28 (9.5%)	20 (7.2%)	48 (8.4%)	33 (23.2%)	29 (26.6%)	62 (24.7%)
PD-L1						
Low	255 (86.4%)	256 (92.1%)	511 (89.2%)	89 (62.7%)	61 (56.0%)	150 (59.8%)
High	40 (13.6%)	22 (7.9%)	62 (10.8%)	53 (37.3%)	48 (44.0%)	101 (40.2%)
Immune activation						
Active*	20 (6.8%)	9 (3.2%)	29 (5.1%)	50 (35.2%)	46 (42.2%)	96 (38.2%)
Inactive/absent†	275 (93.2%)	269 (96.8%)	544 (94.9%)	92 (64.8%)	63 (57.8%)	155 (61.8%)
Subtype						
HER2-positive‡	6 (2.2%)	9 (3.5%)	15 (2.8%)	19 (16.2%)	19 (21.6%)	38 (18.5%)
Luminal A	193 (71%)	175 (67.8%)	368 (69.4%)	26 (22.2%)	18 (20.5%)	44 (21.5%)
Luminal B	70 (25.7%)	72 (27.9%)	142 (26.8%)	37 (31.6%)	27 (30.7%)	64 (31.2%)
Triple-negative	3 (1.1%)	2 (0.8%)	5 (0.9%)	35 (29.9%)	24 (27.3%)	59 (28.8%)
ER status						
Negative	7 (2.6%)	5 (1.9%)	12 (2.3%)	42 (35.3%)	33 (37.1%)	75 (36.1%)
Positive	266 (97.4%)	254 (98.1%)	520 (97.7%)	77 (64.7%)	56 (62.9%)	133 (63.9%)
PgR status						
Negative	41 (15.0%)	48 (18.5%)	89 (16.7%)	59 (49.6%)	48 (53.9%)	107 (51.4%)
Positive	232 (85.0%)	211 (81.5%)	443 (83.3%)	60 (50.4%)	41 (46.1%)	101 (48.6%)
Histological grade						
Grade I	68 (23.1%)	73 (26.3%)	141 (24.6%)	0 (0%)	0 (0%)	0 (0%)
Grade II	227 (76.9%)	205 (73.7%)	432 (75.4%)	10 (7.0%)	9 (8.3%)	19 (7.6%)
Grade III	0 (0%)	0 (0%)	0 (0%)	132 (93.0%)	100 (91.7%)	232 (92.4%)
Endocrine therapy						
No hormone therapy	254 (92.7%)	249 (95.8%)	503 (94.2%)	105 (88.2%)	80 (88.9%)	185 (88.5%)
Hormone therapy	20 (7.3%)	11 (4.2%)	31 (5.8%)	14 (11.8%)	10 (11.1%)	24 (11.5%)
Chemotherapy						
No chemotherapy	274 (100%)	259 (99.6%)	533 (99.8%)	113 (95.0%)	87 (96.7%)	200 (95.7%)
Chemotherapy	0 (0%)	1 (0.4%)	1 (0.2%)	6 (5.0%)	3 (3.3%)	9 (4.3%)
IBTR within 5 years§						
No IBTR	240 (81.4%)	255 (91.7%)	495 (86.4%)	96 (67.6%)	86 (78.9%)	182 (72.5%)
IBTR	37 (12.5%)	7 (2.5%)	44 (7.7%)	27 (19.0%)	8 (7.3%)	35 (13.9%)
Censored	18 (6.1%)	16 (5.8%)	34 (5.9%)	19 (13.4%)	15 (13.8%)	34 (13.5%)
IBTR within 10 years§						
No IBTR	206 (69.8%)	221 (79.5%)	427 (74.5%)	80 (56.3%)	69 (63.3%)	149 (59.4%)
IBTR	54 (18.3%)	23 (8.3%)	77 (13.4%)	33 (23.2%)	10 (9.2%)	43 (17.1%)
Censored	35 (11.9%)	34 (12.2%)	69 (12.0%)	29 (20.4%)	30 (27.5%)	59 (23.5%)

*Defined as TILs ≥10% and PD-L1 and/or PD-1 ≥1%.

†Defined as TILs <10% or TILs ≥10% but PD-L1 and PD-1 <1%.

‡Includes both ER-positive and ER-negative tumors.

§Reported as absolute frequencies rather than cumulative incidences.

ER, estrogen receptor; HER2, human epidermal growth factor receptor 2; IBTR, ipsilateral breast tumor recurrence; PD-1, programmed cell death protein-1 ; PD-L1, programmed death ligand-1 ; PgR, progesterone receptor; RT, radiotherapy; TILs, tumor-infiltrating lymphocytes.

In the low-risk group, high TILs were seen among 108 tumors (18.8%), high PD-1 expression among 48 (8.4%) tumors, and high PD-L1 expression among 62 (10.8%) tumors (table 1). In total, 29 (5.1%) tumors were classified as having an activated immune response. Among low-risk tumors, 12 (2.3%) were ER-negative, 141 (24.6%) of grade I, and 432 (75.4%) of grade II.

### Prognostic effect

In total, 17.2% (13.1–22.5) of patients in the high-risk group and 13.7% (11.1–16.8) of patients in the low-risk group developed an IBTR within 10 years. High-risk tumors with an active immune response had an IBTR rate of 8.4% (4.3–16.1), while high-risk tumors without an active immune infiltrate had an IBTR rate of 22.8% (16.9–30.2). Among high-risk tumors, an activated immune infiltrate was associated with a reduced risk of IBTR in univariable (HR 0.34, 95% CI 0.16 to 0.73, p=0.006) and multivariable (HR 0.33, 95% CI 0.15 to 0.72, p=0.005) analysis (table 2).

**Table 2:** Cox proportional hazard rate regression. Ten-year follow-up of ipsilateral breast tumor recurrence (IBTR) among low-risk and high-risk patients

Variable	Low-risk group (n=573)					High-risk group (n=251)				
	# of IBTR/	Univariable Cox regression	P value	Multivariable Cox regression	P value	# of IBTR/	Univariable Cox regression	P value	Multivariable Cox regression	P value
	# of patients	HR (95% CI)		HR (95% CI)		# of patients	HR (95% CI)		HR (95% CI)	
Immune system										
Not activated	71/544	1.0		1.0		35/155	1.0		1.0	
Activated	6/29	2.0 (0.87 to 4.6)	0.100	1.8 (0.79 to 4.2)	0.159	8/96	0.34 (0.16 to 0.73)	0.006	0.33 (0.15 to 0.72)	0.005
Age (cont.)	77/573	0.98 (0.95 to 1.0)	0.102	–	–	43/251	0.97 (0.94 to 0.99)	0.019	0.97 (0.94 to 0.99)	0.016
Tumor size (cont.)	71/530	1.0 (0.96 to 1.1)	0.739	–	–	39/208	0.98 (0.93 to 1.0)	0.518	–	–
ER										
Negative	4/9	1.0		1.0		11/85	1.0			
Positive	73/561	0.21 (0.08 to 0.56)	0.002	0.17 (0.06 to 0.46)	0.001	32/165	1.4 (0.72 to 2.8)	0.302	–	–
RT										
No	54/295	1.0		1.0		33/142	1.0		1.0	
Yes	23/278	0.41 (0.25 to 0.67)	<0.001	0.40 (0.25 to 0.66)	<0.001	10/109	0.36 (0.18 to 0.74)	0.005	0.42 (0.20 to 0.85)	0.017

ER, estrogen receptor; IBTR, ipsilateral breast tumor recurrence; RT, radiotherapy.

Low-risk tumors with an activated immune infiltrate had a 10-year IBTR rate of 20.9% (10.0–40.7) compared with an IBTR rate of 13.3% (10.7–16.5) among low-risk tumors without an activated immune infiltrate. No significant difference in risk IBTR among low-risk tumors was seen for an activated immune infiltrate (univariable: 2.0, 95% CI 0.87 to 4.6, p=0.100, multivariable: HR 1.8, 95% CI 0.79 to 4.2, p=0.159) compared with not having an activated immune infiltrate (HR 1.0) (table 2. The interaction between immunological activity and risk group was significant in univariable (p=0.005) and multivariable (p=0.007) analysis (table 3).

**Table 3:** Cox proportional hazard rate regression. Ten-year follow-up of ipsilateral breast tumor recurrence (IBTR)

Variables	No. of IBTRs/no. of patients	Univariable Cox regression		Multivariable Cox regression	
		HR (95% CI)	P value	HR (95% CI)	P value
Combination of immune group and risk group					
Not activated, low risk	71/544	1.0		1.0	
Not activated, high risk	35/155	2.1 (1.4 to 3.1)	<0.001	1.9 (1.3 to 2.9)	0.002
Activated, low risk	6/29	2.0 (0.87 to 4.6)	0.105	1.8 (0.76 to 4.0)	0.189
Activated, high risk	8/96	0.68 (0.33 to 1.4)	0.306	0.63 (0.30 to 1.3)	0.217
Interaction			0.005*		0.007*
Age (cont.)	120/824	0.97 (0.95 to 0.99)	0.005	0.97 (0.96 to 0.99)	0.003
Tumor size (cont.)	110/738	1.01 (0.97 to 1.04)	0.761	–	
ER status					
Negative	15/94	1.0		–	
Positive	105/726	0.78 (0.46 to 1.35)	0.376	–	
RT					
No	87/437	1.0		1.0	
Yes	33/387	0.39 (0.26 to 0.58)	<0.001	0.41 (0.27 to 0.61)	<0.001

*Likelihood-ratio test.

ER, estrogen receptor; IBTR, ipsilateral breast tumor recurrence; RT, radiotherapy.

### Benefit from RT

A non-significant benefit from RT was seen among high-risk tumors with an activated immune infiltrate (HR 0.34, 95% CI 0.07 to 1.67, p=0.182), while a significant benefit was observed among high-risk tumors without an activated immune infiltrate (HR 0.40, 95% CI 0.18 to 0.88, p=0.022). Among low-risk tumors with an activated immune infiltrate, the estimates for RT benefit (HR 0.40, 95% CI 0.05 to 3.44, p=0.403) were similar to those of low-risk tumors without an activated immune infiltrate (HR 0.42, 95% CI 0.25 to 0.69, p=0.001).

**Figure 3:**
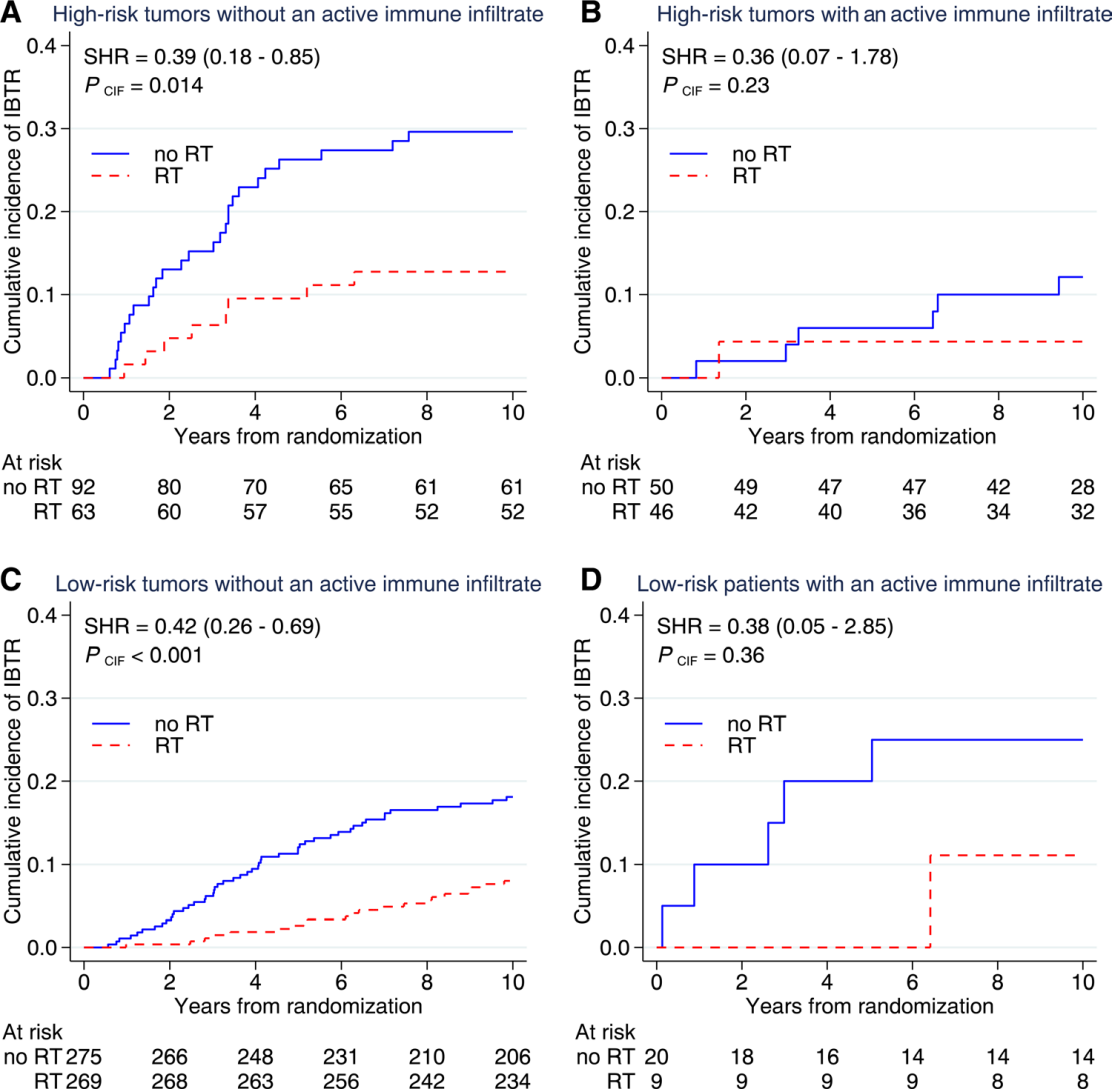
Cumulative incidences among high-risk and low-risk tumors with and without an activated immune response. High-risk tumors were defined as histological grade III or histological grade II with a high Proliferative Index. An activated immune response was defined as tumor-infiltrating lymphocytes ≥10% and ≥1% of lymphocytes positive for programmed cell death protein-1 and/or programmed death ligand-1. IBTR, ipsilateral breast tumor recurrence; RT, radiotherapy; SHR, subdistribution hazard ratio; CIF, cumulative incidence function.

Figure 3 illustrates the cumulative incidences depending on RT, immune activation, and tumor-intrinsic risk group. High-risk tumors with an activated immune response had a 10-year incidence of IBTR of 12.1% (5.6–25.0) without RT and 4.4% (1.1–16.3) with RT. This can be contrasted against high-risk tumors with an absent immune response, where the 10-year incidence of IBTR was 29.6% (21.4–40.2) without RT and 12.8% (6.6–23.9) with RT. Low-risk tumors with an activated immune response had a 10-year IBTR incidence of 25.0% (11.2–50.0) without RT and 11.1% (1.6–56.7) with RT, while low-risk tumors without an activated immune infiltrate had a 10-year incidence of IBTR of 18.1% (14.0–23.3) without RT and 8.4% (5.6–12.5) with RT.

### Exploratory analyses

As a post hoc exploratory analysis, we compared the high-risk groups with TILs 10–49% and 50–100% to investigate a potential dose-response relationship. Unirradiated patients with TILs 10–49% had a 10-year cumulative IBTR incidence of 15% (0.07–0.29). Unirradiated patients with TILs 50–100% had a lower, but not significantly different, cumulative IBTR incidence of 13% (0.05–0.31) (online supplemental figure S2).

Finally, to verify the stability of the results, we re-ran the main analyses excluding patients treated with systemic therapy. The findings remained stable (online supplemental tables S7 and S8).

## Discussion

The integration of immunological and tumor-intrinsic factors enables the successful stratification of high-risk tumors regarding the risk of IBTR. The present study shows that this can generally be achieved with variables already used in the clinic and that aggressive tumors, including luminal subtypes, with an active immune response, have a low risk of IBTR even without RT boost and systemic therapy. High-risk tumors with activated immune infiltrates had the lowest rates of IBTR, highlighting the possibility to de-intensify locoregional treatment.

Research on biological predictors to inform RT de-escalation is ongoing. The recently published POLAR classifier may identify ER-positive HER2-negative tumors suited for RT omission.^32^ Genes associated with proliferation were associated with an increased risk of locoregional recurrence. The current study highlights a parallel de-escalation pathway on the opposite side of the proliferation spectrum, where traditionally regarded high-risk tumors, irrespective of ER status, can be downgraded if they benefit from an activated antitumor immune response. Patients with high-risk tumors with an activated immune infiltrate had a relatively low risk of IBTR unirradiated (12.1%) and irradiated (4.4%), despite standard RT (ie, without an RT boost) and a low frequency of systemic therapy. These tumors may have a delayed local and systemic dissemination preoperatively and inhibited regrowth of postoperative residual disease, reducing the need for RT treatment. With modern systemic treatment, the 10-year incidence of IBTR may be below 10% without RT. The findings align with another recent study showing that young patients with triple-negative breast cancer and high TILs have a surprisingly good prognosis without adjuvant therapy.^5^ Immune-responsive tumors with very high TIL levels (eg, ≥50%) may represent an RT omission group, while moderately increased TILs (eg, 10–49%) could justify RT boost omission. Although we hypothesize that low-risk tumors are best stratified for treatment de-escalation using proliferation measurements, the role of the immune response among these is not fully understood. We and others have previously shown that global measures of immune activation confer a favorable prognosis only among high-risk tumors.^14 33 34^ However, it cannot be excluded that activation of certain immune response subcomponents may still benefit low-risk tumors. For example, the humoral immune system may reduce the recurrence risk in luminal tumors,^35^ which conforms with findings of B-cell-related genes in POLAR predicting a favorable prognosis.^32^

We have previously shown that integrating tumor-intrinsic factors in the assessment of immunological biomarkers can improve the identification of high-risk tumors with different needs for RT. 14 CD8+T cells, the primary effector cell of antitumor immunity, recognize and are activated by neoantigens generated by tumor mutations.^7^ Therefore, tumor-intrinsic factors that correlate with proliferation and tumor mutational burden (TMB) may inform the likelihood that an immune infiltrate represents an active antitumoral immune response.^34^ Histological grade correlates with proliferation and TMB,^36^ and we hypothesized that histological grade might capture tumor-intrinsic qualities necessary to understand the biological influence of an immune infiltrate. In the SweBCG91RT cohort, PD-1 and/or PD-L1 were expressed by the majority of high-risk tumors with high TILs. Conversely, high TILs were less frequently associated with PD-1/PD-L1 expression among low-risk tumors, indicating that an immune infiltrate in these tumors has other biological implications. This is supported by studies showing an absent or unfavorable prognostic effect of immune infiltrates in low-risk tumors.^28–30^

Despite the overwhelming focus on triple-negative and HER2-positive subtypes in TILs research, most tumors with TILs are ER-positive.^11^ However, the lack of understanding of how TILs influence tumor progression among ER-positive tumors has prevented TILs from being used as a biomarker in this group.^11^ A better understanding may enable the implementation of immunotherapy on a subset of immunogenic ER-positive tumors.^11^ We found that the majority of tumors classified as high-risk, and deriving a significant benefit from an activated immune infiltrate, were ER-positive (63.9%), echoing the unmet potential for using TILs as a biomarker among these tumors.^11^ The International Immuno-Oncology Biomarker Working Group highlights the need for more research on TILs among ER-positive subtypes, stratifying analyses by luminal A and luminal B.^11^ However, our results indicate that there may exist heterogeneity within these subtypes, as all subtypes were relatively equally represented in the high-risk group. Our findings add a layer of complexity to previous observations^37^ by suggesting that it may not be subtype, but instead characteristics that can in part be approximated by subtype, that predict the biological influence of an immune infiltrate. These findings align with a previous study where luminal B tumors with aggressive tumor characteristics demonstrated immunotherapy responsiveness.^19^ We believe additional measures of tumor aggressiveness, such as histological grade or proliferation, are needed to accurately predict the implications of an immune infiltrate, particularly in the case of luminal B tumors, where the degree of proliferation can vary considerably.^18^ It remains to be determined if tumor-intrinsic characteristics predict immune responsiveness also in cohorts dominated by non-luminal subtypes.

Assessments of the PD-1/PD-L1 pathway are today used as biomarkers for immunotherapy in metastatic triple-negative breast cancer.^38^ Expression is associated with an improved prognosis,^39^ despite the inherent immunosuppressive effects, likely due to its association with an active immune response. PD-1 is expressed on activated T cells,^40^ and PD-L1, expressed by a wide range of cells, for example, T-regulatory cells and tumor cells, is upregulated by inflammatory signaling.^41^ Furthermore, measurements of the PD-1/PD-L1 pathway provide independent information in addition to TILs.^26^ For this reason, we used high TILs combined with the expression of PD-1 or PD-L1 to characterize an active immune response.

We used histological grade and a gene expression-based proliferation signature as tumor-intrinsic predictors of immune responsiveness.^42^ However, additional tumor characteristics should be considered. One such factor is HER2 status, which has emerged as a key biomarker in breast cancer. HER2-positive tumors are considered immunogenic, and anti-HER2 therapy functions partly by inducing an antitumoral immune response.^42 43^ Unfortunately, due to the low number of HER2-positive tumors and lack of anti-HER2 therapy, we did not try to answer whether HER2 positivity should be included as a variable predictive of immune responsiveness. Therefore, future studies should investigate whether HER2 positivity and additional tumor-intrinsic characteristics, such as TMB, provide independent information beyond histological grade and tumor proliferation regarding immune responsiveness.

There are several weaknesses in the present study. First, our question involved post hoc analyses of subgroups, which reduces the power and should be viewed as hypothesis-generating. The low-risk group with an active immune infiltrate was small, why findings pertaining to this group should be interpreted cautiously. Second, many patients would have received a different therapy regimen had they been diagnosed today. No patients in the SweBCG91RT study received an RT boost, although some of them would be recommended a boost in the current situation. In addition, few patients received systemic treatment, which would likely have significantly reduced the risk of IBTR.^44^ Furthermore, systemic anti-HER2 therapy and chemotherapy treatment would probably have produced a differential benefit for patients, with highly proliferative immunogenic tumors showing the best response.^45 46^ While the above limits the generalizability of our findings, it also indicates that modern treatment would preferentially have reduced the risk of IBTR in the high-risk immunogenic group, further supporting de-escalation of RT as a valid strategy for these patients. Nevertheless, our findings apply primarily to a setting free of adjuvant systemic therapy. The high cut-off used to classify grade II tumors as high-risk resulted in only a minority of these tumors being classified as such and did not allow for thoroughly investigating immune responsiveness along the spectrum of tumor aggressiveness among grade II tumors. We used this high cut-off based on the hypothesis that most grade II tumors should be classified as low-risk and to avoid dilution of the hypothesized effect size of the high-risk group. We did not test additional cut-offs and cannot determine the proportion of grade II tumors likely to benefit from an immune infiltrate. This should be investigated in future studies. However, the finding that grade II tumors resemble grade I tumors more than grade III tumors in terms of a gene expression signature designed to measure immune responsiveness indicates that most should be classified as low-risk. Finally, the use of TMAs may miss tumor heterogeneity. Previous studies have shown that around three TMAs may be sufficient to categorize a tumor as having high or low TILs.^47^ Since we used four TMAs to assess the activity of the PD-1/PD-L1 axis, we believe the risk of missing tumor heterogeneity is reduced, although not eliminated.

There is a large variation in analytical sensitivity between different PD-L1 immunohistochemistry (IHC) assays, with SP142, used in the present study, shown to have poor sensitivity.^48^ Consequently, some tumors classified as PD-L1 negative were likely false negatives, indicating that findings should be interpreted cautiously. The optimal IHC assay identifying immunogenic tumors would preferably have a higher sensitivity than SP142. Furthermore, a potential added value to TILs of additional immunological markers, such as PD-1/PD-L1 expression,^25^ may be partly or entirely explained by an association with even higher TILs. Therefore, assessing TILs as a continuous variable on whole sections may be a sufficiently robust measurement to identify tumors with different immune activation degrees and tailor therapy accordingly.

In conclusion, high-risk tumors with an activated immune infiltrate have a surprisingly good prognosis in terms of local recurrences. The risk reduction regarding IBTR conferred by an activated immune infiltrate among these tumors may be comparable to treatment with RT. Therefore, we hypothesize that these patients do well with the de-escalation of RT treatment. Our findings likely apply to low-risk early breast cancer cohorts dominated by ER-positive tumors.

10.1136/jitc-2022-006618.supp2 Supplementary data

## Data Availability

Data are available upon reasonable request. Gene expression data from the SweBCG91RT cohort has been made available in the Gene Expression Omnibus database (GSE119295). However, due to regulations of the ethical review board and of laws related to patient privacy, all clinical information has not been made publicly available. The IHC data used in this study is/are available from the corresponding author upon reasonable request.
