# Supplementary material for: Combining histological grade, TILs, and the PD-1/PD-L1 pathway to identify immunogenic tumors and de-escalate radiotherapy in early breast cancer: a secondary analysis of a randomized clinical trial
Source: J Immunother Cancer. 2023 May 19;11(5):e006618. doi: 10.1136/jitc-2022-006618 (PMC10201214; doi:10.1136/jitc-2022-006618)
Supplement: Supplementary data [file jitc-2022-006618supp003.pdf]

ONCOLOGICAL CENTER

for the Southern Health Care Region

SB 91: A

Breast conserving treatment with or without postoperative radiotherapy in breast cancer stage T 1-2 (<30 mm), pN0, M0

A national multi-center study

Prepared by

Sydsvenska Breast Cancer Group

1990

Replaces national breast cancer treatment program for Stage I, 1982

PROGRAM DESCRIPTION - STUDY SB 91: A

1. OBJECTIVE

1.1 Primary objective

The study involves a comparison between standardized sector resection and axillary lymph node dissection with or without routine postoperative radiotherapy against residual breast parenchyma in invasive breast cancer stage T 1-2 (max. Diam 30 mm), pN0, M0.

The main aim is to test the hypothesis that local tumor control is achieved without post-operative radiotherapy provided that the surgical procedure is well standardized and aims to optimize the conditions for local radicality.

1.2 Secondary objectives

- Is there a difference in mortality between the two treatment options?

- Is local recurrence mainly due to lack of surgical radicality or due to progression of multicentric changes in other parts of the breast?

- Can patients with high risk of local recurrence already be identified during primary treatment and then selected for post-operative radiation therapy?

- Does local radiation treatment have any effect on the incidence of cancer in the ipsilateral and the contralateral breast?

2. Ethics

2.1 The study has been reviewed and approved by the Research Ethics Committee at Lund University.

2.2 The patient shall be adequately informed of the nature of the disease and current treatment options.

2.3 A template for oral information is attached to the protocol.

2.4 The study has been prepared in accordance with the principles of the Helsinki Declaration 1964.

3. DIAGNOSIS

3.1 Diagnosis of breast cancer is not regulated in this protocol but follows the usual principles. Triple diagnostics (clinical examination, mammography and cytology) should always be performed.

3.2 If required, excision biopsy should, if possible, be carried out in the form of sector resection.

3.3 Biopsy of non-palpable breast changes requires access to the preparation X-ray.

3.4 Tumor tissue tests for steroid receptor determination and DNA analysis should be routinely performed if the tumor size allows such sampling. However, these analyzes are not required for participation in the study.

3.5 In primary surgery, pulmonary X-ray and liver status (S-Car, S-ALP, S-ASAT, S-ALAT, S-GT, S-Ca) are recommended. Further metastatic diagnostics is performed only if clinical or laboratory signs for distant metastasis are present.

4 Patient selection

4.1 Participating clinics should aim to include all patients who fulfill the criteria for randomization. Randomization can occur when all inclusion criteria are met. This means that a definitive result from the histopathological analysis must be present.

4.2 A total of 1100 patients (in the whole country) should be randomized into the study before the inclusion is stopped.

4.3 Patients with tumor stage T1-2 N0 who do not fulfill the eligibility criteria according to 4.4 should be treated according to the current guidelines.

4.4 Eligibility criteria

4.4.1 Female with invasive primary breast cancer.

4.4.2 The patient's age should not exceed 75 years on the day of the operation. No lower age limit.

4.4.3 No signs of distant metastases during preoperative complete clinical examination.

4.4.4 If the tumor is radiologically visible, preoperative mammography should show a tumor diameter of maximum 30 mm. The mammography must not show multiple tumors in multiple quadrants and not microcalcifications beyond the sector which can be safely excised with the tumor.

4.4.5 The tumor should be excised with a sector resection as primary surgery procedure or as a resection after diagnostic biopsy.

4.4.6 Radical excision according to histopathological assessment. If the radicality is uncertain, a completing radical excision is necessary.

4.4.7 Histopathological analysis should show tumor radicality and abscence of signs of multifocal cancer which includes invasive cancer or cancer in situ further than 2 cm away from the periphery of the primary tumor.

4.4.8 No signs of lymph node metastases in histopathological analysis after axillary lymph node dissection. At least 5 lymph nodes should be analyzed.

4.4.9 The relationship between tumor and breast size should allow local radicality with an acceptable cosmetic result.

4.4.10 The patient is informed and accepts sector resection with or without radiotherapy as definitive treatment.

4.4.11 It is possible to follow the patient and the patient should not suffer from another serious illness, such as severe dementia, a severe psychological disorder or drug addiction problems.

4.4.12 The patient can participate in the radiotherapy treatment.

5. Randomization

5.1 Patients who meet all of the eligibility criteria according to 4.4.

5.2 Randomization is done by phone 046 - 17 75 60 to Oncologic Center, Lasarettet, Lund.

5.3 Randomization occurs directly after the results of the histopathological examination are definitive.

5.4 Stratification takes place for participating clinics and if the tumor is detected by screening or not.

5.5 Randomization occurs to either of the two treatment options:

- Postoperative radiotherapy against the operated breast.

- No postoperative radiotherapy.

5.6 Randomization occurs simultaneously for adjuvant treatment in accordance with study protocol SB 91: B

5.7 Patients randomized to radiotherapy are immediately referred to oncologic clinic with information that the patient participates in the study.

5.8 Breast cancer application form is submitted immediately after randomization to the Department of Oncology Center.

6. POST-OPERATIVE TREATMENT

6.1 Radiotherapy

Postoperative radiotherapy is initiated as soon as the wound has healed and is administered according to the technical description in the national guidelines.

6.2 Adjuvant treatment

Premenopausal patients should receive adjuvant treatment according to SB 91: B (separate study). Postmenopausal women with tumor size 21-30 mm should also be included in SB II: 2 and be randomized to Tamoxifen for 2 and 5 years respectively.

6.3 Treatment of local recurrence

Treatment of local recurrences in remaining breast parenchyma is not regulated in this protocol and should be determined in a joint consultation between the patient and the treating physician. In the group treated with radiotherapy, mastectomy is recommended. In limited recurrences in the breast among patients not treated with radiotherapy, a new local excision with adjuvant radiotherapy should be considered.

6.4 Treatment of regional recurrences and distal metastases

This treatment is not regulated in this protocol.

7. FOLLOW-UP

7.1 All patients are followed by clinical examination every six months for two years, then annually to 5 years.

7.2 Clinical mammography is performed annually for 10 years.

7.3 After 10 years, patients are referred to general health examination with mammography.

7.4 Results after 10 years are requested.

7.5 All randomized patients should be checked in the above manner regardless of whether they completed treatment or not.

7.6 In addition, additional controls may be considered, for example, in case patients are included in another study.

7.7 Results of each control are reported on the form "Follow-up of breast cancer patients".

8. DEFINITION OF CLINICAL EVENTS

8.1 Local recurrence

Registration of recurrence in the treated breast constitutes the primary purpose of the study.

All cytologically or histopathologically verified relapses in remaining breast parenchyma or in cutis and subcutis adjacent to the breast are classified as local relapse.

When local recurrence occurs, they should, if possible, be classified into either of the following categories:

- recurrence in previously operated areas
- recurrence / new tumor in the breast parenchyma outside of the previously operated area
- recurrence in intramammaric lymph node or as emboli in lymph vessels
- recurrence outside of the breast parenchyma, i.e. in cutis or subcutis

Subsequently diagnosed lymph node metastases, for example processus axillaris, are classified as regional recurrence.

If there is difficulty in clearly referring a patient to one of the four above-mentioned groups, the case is referred to the project group for classification without the knowledge of the treatment group.

8.2 Death

In the event of death, autopsy is sought. The pathologic departments should be advised to pay special attention to whether they receive autopsy for breast cancer surgery on the basis of breast cancer.

When patients randomized to the study die, one should seek classification in one of the following categories:

- death due to breast cancer

- death with residual breast cancer but with other major cause of death.

- death without signs of breast cancer relapse

If there is difficulty in clearly referring a patient to one of these categories, the case is referred to the project group for classification without the knowledge of the treatment group.

9 EVALUATION AND STATISTICS

9.1 The endpoints for the analysis are local recurrence, new primary tumor or death. Analysis of relapsed survival and total survival should be performed for all randomized patients.

9.2 Material Size

To determine the overall size of the study, it is assumed that the expected local recurrence rate after 5 years is 5% in the radiotherapy group. Below is the required total size of the study for one or two-sided tests on the significance level 0.05 with the probability 0.80 to detect expected local recurrence rates in the non-irradiated group of 7%, 9%, 10% and 11%.

Expected Share Required mtrl size (total)

local recurrence (alpha = 0.05, beta = 0.80)

+ RT	-RT	
	Two-sided test	One-sided test
0,05	0,07<br>4400	3500
0,05	0,09<br>1280	1000
0,05	0,10<br>870	680

0,05	0,11<br>640	500

When it can be ruled out in advance that the local rate of return is lower in the non-radiotherapy group, the study should be dimensioned based on a one-sided test. With 1000 patients, one can then detect a difference in the expected frequency from 0.05 to 0.09 with the probability of 0.8. With correction for 5% loss, the material size will then reach 1100 patients.

9.3 Interim Assessment

Interim evaluation should be done when 600 patients are randomized. The study should be discontinued if there is a significantly higher mortality ($p < 0.05$) in either group or if a life-table estimate of local recurrence rate after three years is above 15% for the non-radiated group.

Cancellation decisions take place after the meeting with the project team.

9.4 Management alignment

This study is management-oriented, which means that patients are analyzed in the group to which they have been randomized, regardless of whether treatment is completed or not.

9.5 Analysis

The statistical processing will be done with unidentified treatment groups.

10. ADMINISTRATION

10.1 The study is planned in consultation with the National Association against Cancer Planning Group for Breast Cancer. This group, as well as the work committee for the study, constitutes a national management team which is responsible for long-term continuity within the study and initiates the scientific evaluations. The management team coordinates locally initiated sub-projects.

10.2 The work committee for the study consists of Lars Holmberg, Uppsala; Stefan Rydén, Ängelholm; Lars-Erik Rutqvist, Stockholm; John Carstensson, Linköping and by a contact person for each participating region.

The work committee is responsible for the practical management of the study and is responsible for the management team. The work committee manages ongoing organizational

and scientific issues within the study, monitoring data quality and conducting secretarial functions.

10.3 The study forum for information, discussion and coordination is a project group. This consists of a responsible person from each participating clinic. The management team is represented in the project committee of the work committee. The project group discusses all fundamentally important questions in the study.

10.4 Randomization and dispatch and collection of forms and data readings take place at the Oncological Center, Lasaret, Lund, which is responsible for validation and monitoring. A secretary directly under the work committee is responsible for contacts between the work committee and the regional oncology centers, recurrent compilations of fact quality control and reports.

11. PUBLICATIONS

11.1 Each publication based on the clinical material is based on all participating clinics and oncological centers. The presentation will take place throughout the project group and management team name. To each publication, an addendum is attached clearly indicating who have been actively involved in the study's implementation at the various clinics, processing and completion of the script.

11.2 The co-authorship of publications analyzing special aspects such as histopathology, mammography, DNA content or hormone receptor content- will be discussed jointly between the participants in the project. In principle, the Medical Association's guidelines must be followed (Läkartidningen 79: 2454-2455, 1982).

11.3 Each clinic participating in the study may use the material for regional or local information in the form of lectures. However, it should always be stated which clinics participated in the survey.

11.4 Local-initiated sub-projects within the study are published by the respective project managers.

TEMPLATE FOR PUBLIC PATIENT INFORMATION

Background

This study is based on the belief that mortality will not differ between the radiation treated and the non-radiated group. On the other hand, the incidence of undiscovered multicentric changes may cause a higher local rate of recurrence in the non-radiated group. The local recidivism is most likely to be detected early in the annual mammography controls. Renewed local excision followed by radiation therapy against residual breast parenchyma should therefore be possible in most cases. In view of this, the disadvantages of routine postoperative radiation treatment may be greater than the benefits.

Discussing these considerations in detail with the individual patient would be difficult and often contrary to the Helsinki Declaration's position that information should not be given if it injures the patient.

Previous experience of oncological trials showed that it is difficult properly to inform patients about the randomization process and to putt hem at an open choice in the form of two treatment options without support or recommendation from treating physicians.

The patient should be informed that a study is in progress according to the following guidelines and that the patient will be included in the study if they accept the treatment of the doctor. The information should of course be adapted to any participating clinic according to suggestions and requirements from local or regional ethics committee.

Information

You are, as we now know, in a favorable disease state with limited tumor without spreading to the lymph nodes in the armpit. Therefore, we do not consider the treatment to be improved if the entire breast is removed but instead propose that only the part of the breast containing tumor is removed.

After the surgery there are two different treatment options. One means that radiation treatment is given to the breast for about 5 weeks. On the other hand, radiation therapy is only provided

if you develop tumor recurrence in the breast. You must first do a renewed surgery, but in the number of cases your chest can be preserved.

Nothing suggests that the different finishes differ in terms of the most important outcome: the chance of lasting cure. The difference is in local effects. After radiation therapy, there is a certain risk of radiation effects in the short and long term. In surgery without radiation treatment there is a slightly increased risk of local relapses.

Which postoperative treatment is best is an undisclosed question. We therefore propose that we determine what treatment should be given as part of an investigation.

This is then determined according to a predetermined list. You are in full right to refuse participation or to cancel participation in the survey and may then choose the treatment you wish. Parts of the journal will be processed without the possibility of identifying that participation.
