# Supplementary material for: Combining histological grade, TILs, and the PD-1/PD-L1 pathway to identify immunogenic tumors and de-escalate radiotherapy in early breast cancer: a secondary analysis of a randomized clinical trial
Source: J Immunother Cancer. 2023 May 19;11(5):e006618. doi: 10.1136/jitc-2022-006618 (PMC10201214; doi:10.1136/jitc-2022-006618)
Supplement: Supplementary data [file jitc-2022-006618supp001.pdf]

Supplement

Table of contents

Figure S1. Association of Proliferative index with histological grade 3

Figure S2. Cumulative incidence of IBTR in the high-risk group with increasing levels of TILs 4

Table S1. Comparison of included versus excluded patients 5

Table S2. Cox regression regarding IBTR with 5-year follow-up among all patients..............................6

Table S3. Cox regression regarding IBTR with 5-year follow-up among low-risk tumors........................7

Table S4. Cox regression regarding IBTR with 5-year follow-up among high-risk tumors.......................8

Table S5. Estrogen receptor expression levels in the high- and low-risk groups 9

Table S6. Levels of TILs among patients with TILs ≥10% with and without PD-1/PD-L1 expression 10

Table S7. Cox proportional hazard rate regression excluding those with systemic treatment.............11

Table S8. Cox proportional hazard rate regression among low- and high-risk patients excluding those with systemic treatment 12

Examples of positive and negative staining of PD-1/PD-L1 13

Staining protocols for PD-1 and PD-L1 stainings 14

Figure S1. Association of Proliferative Index with histological grade in the SweBCG91RT cohort

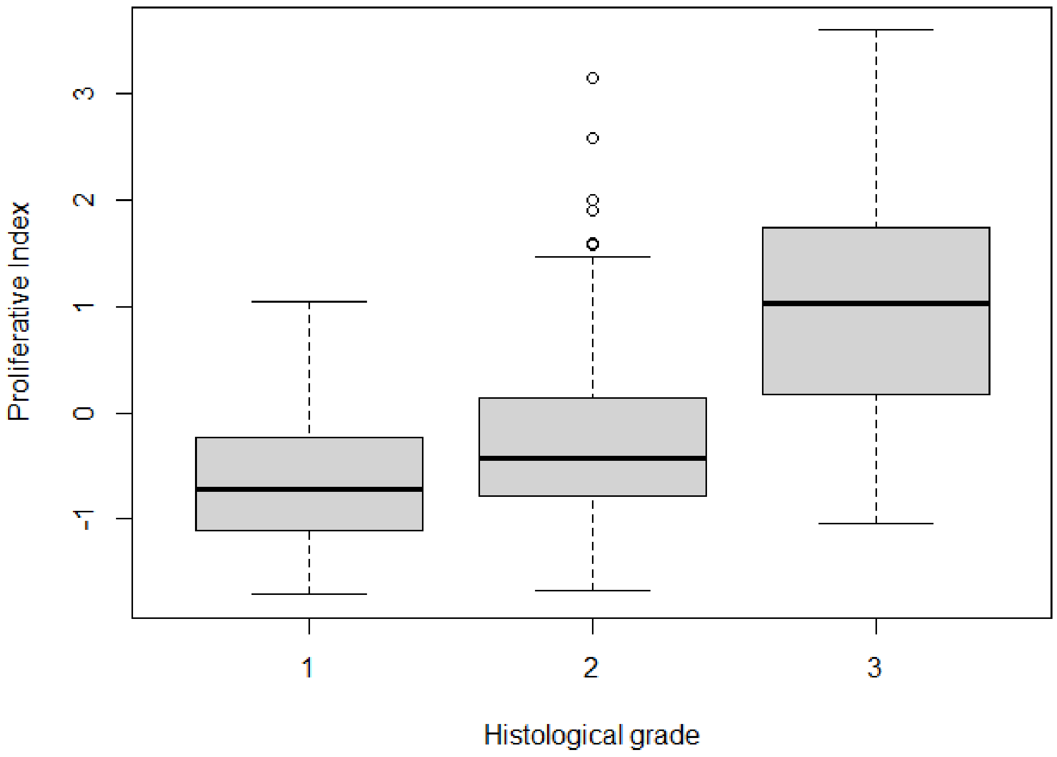

Figure S2. Cumulative incidence of IBTR in the high-risk group with increasing levels of TILs

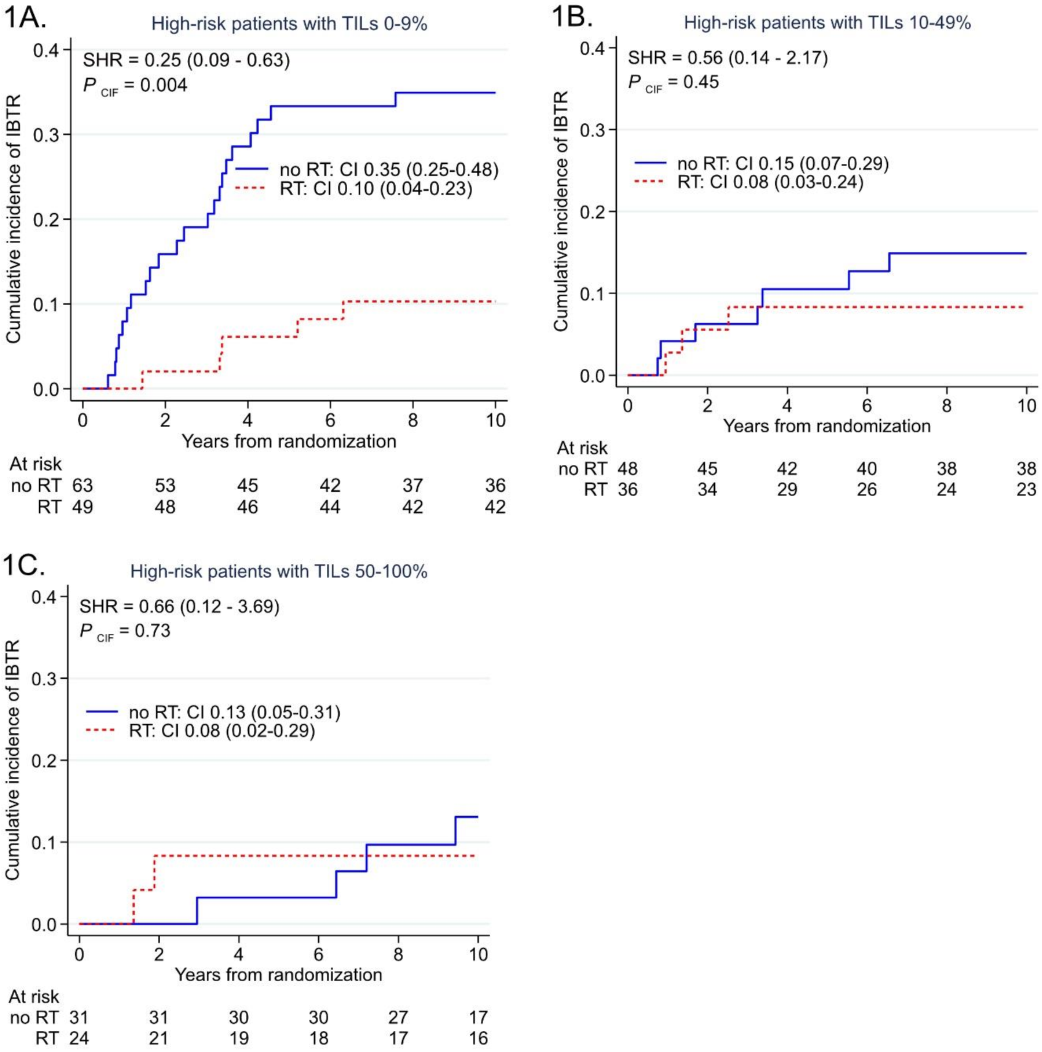

RT= Radiotherapy. TILs= Tumor-infiltrating lymphocytes. SHR= Subhazard ratio for RT versus no RT. CI= Cumulative incidence.

Table S1. Comparison of included versus excluded patients

Variables	Included	Excluded	P
Age median	59	61	
Age mean	58.47	59.50	0.060
Age min	31	32	
Age max	78	78	
RT treated no	437 (53%)	178 (49%)	0.21
RT treated yes	387 (47%)	185 (51%)	
1-10 mm	183 (22%)	126 (35%)	<0.001
11-15 mm	419 (51%)	162 (45%)	
16-20 mm	95 (12%)	30 (8%)	
>20 mm	122 (15%)	41 (11%)	
Size missing	5	4	
Histological grade I	141 (17%)	7 (5%)	0.013
Histological grade II	451 (55%)	122 (91%)	
Histological grade III	232 (28%)	5 (4%)	
Histological grade missing	0	229	
Systemic treatment yes	681 (92%)	336 (93%)	0.64
Systemic treatment no	62 (8%)	27 (7%)	

Table S2. Cox proportional hazard rate regression. 5-year follow-up of local recurrence (IBTR).

Variable	# of IBTR/ # of patients	Univariable Cox regression		Multivariable Cox regression	
		HR (95% CI)	P	HR (95% CI)	P
Combination of Immune system and Risk group					
Not activated, low risk	40/544	1.0		1.0	
Not activated, high risk	30/155	3.0 (1.9-4.9)	<0.001	2.7 (1.7-4.4)	<0.001
Activated, low risk	4/29	2.2 (0.81-6.3)	0.121	1.8 (0.66-5.2)	0.242
Activated, high risk	5/96	0.75 (0.30-1.9)	0.553	0.69 (0.27-1.8)	0.439
Interaction			0.005*		0.010*
Age (cont.)	79/824	0.97 (0.95-0.99)	0.014	0.97 (0.95-0.99)	0.013
Tumor size (cont.)	71/738	1.02 (0.98-1.06)	0.380	-	
ER					
Negative	12/94	1.0		-	
Positive	67/726	0.63 (0.34-1.17)	0.147	-	
RT					
No	64/437	1.0		1.0	
Yes	15/387	0.25 (0.14-0.44)	<0.001	0.27 (0.15-0.47)	<0.001

*Likelihood-ratio test.

Table S3. Cox proportional hazard rate regression. 5-year follow-up of local recurrence (IBTR) among low risk-patients (n=573)

Variable	# of IBTR/ # of patients	Univariable Cox regression		Multivariable Cox regression	
		HR (95% CI)	P	HR (95% CI)	P
Immune system					
Not activated	40/544	1.0		1.0	
Activated	4/29	2.3 (0.81-6.3)	0.121	1.9 (0.69-5.4)	0.210
Age (cont.)	44/573	0.99 (0.96-1.0)	0.582	-	-
Tumor size (cont.)		1.0 (0.95-1.1)	0.732	-	-
ER					
Negative	4/9	1.0		1.0	
Positive	40/561	0.12 (0.04-0.33)	<0.001	0.09 (0.03-0.25)	<0.001
RT*					
No	37/295	1.0		1.0	
Yes	7/278	0.19 (0.08-0.42)	<0.001	0.18 (0.08-0.41)	<0.001

Table S4.

Cox proportional hazard rate regression. 5-year follow-up of local recurrence (IBTR) among high-risk patients (n=251)

Variable	# of IBTR/ # of patients	Univariable Cox regression		Multivariable Cox regression	
		HR (95% CI)	P	HR (95% CI)	P
Immune system					
Not activated	30/155	1.0		1.0	
Activated	5/96	0.25 (0.10-0.65)	0.005	0.25 (0.10-0.64)	0.004
Age (cont.)	35/251	0.95 (0.92-0.99)	0.006	0.96 (0.93-0.99)	0.005
Tumor size (cont.)	31/208	1.0 (0.94-1.1)	0.908	-	-
ER					
Negative	8/85	1.0			
Positive	27/165	1.6 (9.75-3.64)	0.212	-	-
RT*					
No	27/142	1.0		1.0	
Yes	8/109	0.36 (0.16-0.79)	0.011	0.44 (0.20-0.97)	0.041

Table S5. Estrogen receptor expression levels in the high- and low-risk groups

Risk group (column percent/row percent)	0%	1-9%	10-49	50-74%	75-100%	P*
Low-risk	12 (13.6%/2.2%)	4 (80%/0.7%)	3 (37.5%/0.6%)	5 (62.5%/0.9%)	514 (80.9%/95.5%)	
High-risk	76 (86.4%/36.9%)	1 (20%/0.5%)	5 (62.5%/2.4%)	3 (37.5%/1.5%)	121 (19.1%/58.7%)	<0.001

*Fisher's exact test

More than half of the tumors in the high-risk category demonstrated a high estrogen receptor expression. Around one in five (19.1%) strongly ER-positive tumors may be hypothesized to be immune responsive based on this data, which may indicate that subtype alone is insufficient to identify these tumors.

Table S6. Levels of TILs among patients with TILs ≥10% with and without PD-1/PD-L1 expression

Groups		TILs 10-49%	TILs 50-74%	TILs 75-100%	P*
PD-1/PD-L1 expression	No	97 (89.0%)	10 (9.2%)	2 (1.8%)	<0.001
	Yes	66 (58.9%)	29 (25.9%)	17 (15.2%)	

*Fisher's exact test

PD-1/PD-L1 expression was defined as the expression of PD-1 or PD-L1 in ≥1% of lymphocytes in at least one TMA. Among tumors with TILs ≥10%, PD-1/PD-L1 expression was associated with higher TILs levels

Table S7. Cox proportional hazard rate regression of ipsilateral breast tumor recurrence (IBTR) with

			Univariable Cox regression		Multivariable Cox regression	
**Variables**		**No. of IBTRs/No. of patients**	**HR (95% CI)**	**P**	**HR (95% CI)**	**P**
**Combination of immune group and risk group**						
	Not activated, low risk	70/512	1.0		1.0	
	Not activated, high risk	33/132	2.2 (1.5-3.4)	<0.001	2.1 (1.4-3.2)	<0.001
	Activated, low risk	6/27	2.1 (0.91-4.8)	0.080	1.9 (0.81-4.3)	0.144
	Activated, high risk	7/79	0.69 (0.32-1.5)	0.349	0.65 (0.30-1.4)	0.285
	Interaction			0.002*		0.005*
**Age (cont.)**		116/750	0.97 (0.95-0.99)	0.005	0.97 (0.96-0.99)	0.005
**Tumor size (cont.)**		106/676	1.02 (0.98-1.06)	0.403	-	
**ER status**	Negative	13/72	1.0		-	
	Positive	103/674	0.72 (0.40-1.28)	0.259	-	
**RT#**	No	85/391	1.0		1.0	
	Yes	31/359	0.36 (0.24-0.54)	<0.001	0.38 (0.25-0.57)	<0.001

10-year follow-up excluding patients with systemic treatment (n=74)

*Likelihood-ratio test.

Table S8. Cox proportional hazard rate regression of ipsilateral breast tumor recurrence (IBTR) 10-year follow-up among low-risk- and high-risk patients excluding patients with systemic treatment (n=74)

	Low-risk group (n=539)					High-risk group (n=211)				
	# of IBTR/	Univariable Cox regression		Multivariable Cox regression		# of IBTR/	Univariable Cox regression		Multivariable Cox regression	
Variable	# of patients	HR (95% CI)	P	HR (95% CI)	P	# of patients	HR (95% CI)	P	HR (95% CI)	P
Immune system										
Not activated	70/512	1.0		1.0		33/132	1.0		1.0	
Activated	6/27	2.1 (0.93-4.9)	0.075	2.0 (0.85-4.5)	0.115	7/79	0.32 (0.14-0.72)	0.006	0.32 (0.14-0.74)	0.007
Age (cont.)	76/539	0.98 (0.96-1.0)	0.119	-	-	40/211	0.96 (0.94-0.99)	0.018	0.97 (0.94-1.0)	0.021
Tumor size (cont.)	70/498	1.0 (0.97-1.1)	0.378	-	-	36/178	0.99 (0.93-1.1)	0.730	-	-
ER										
Negative	3/8	1.0		1.0		10/64	1.0			
Positive	73/528	0.29 (0.09-0.91)	0.034	0.23 (0.07-0.73)	0.012	30/146	1.2 (0.60-2.5)	0.580	-	-
RT[#]										
No	53/274	1.0		1.0		32/117	1.0		1.0	
Yes	23/265	0.41 (0.25-0.66)	<0.001	0.40 (0.25-0.66)	<0.001	8/94	0.29 (0.13-0.62)	0.002	0.32 (0.15-0.69)	0.004

Stainings of PD-1/PD-L1

Negative staining

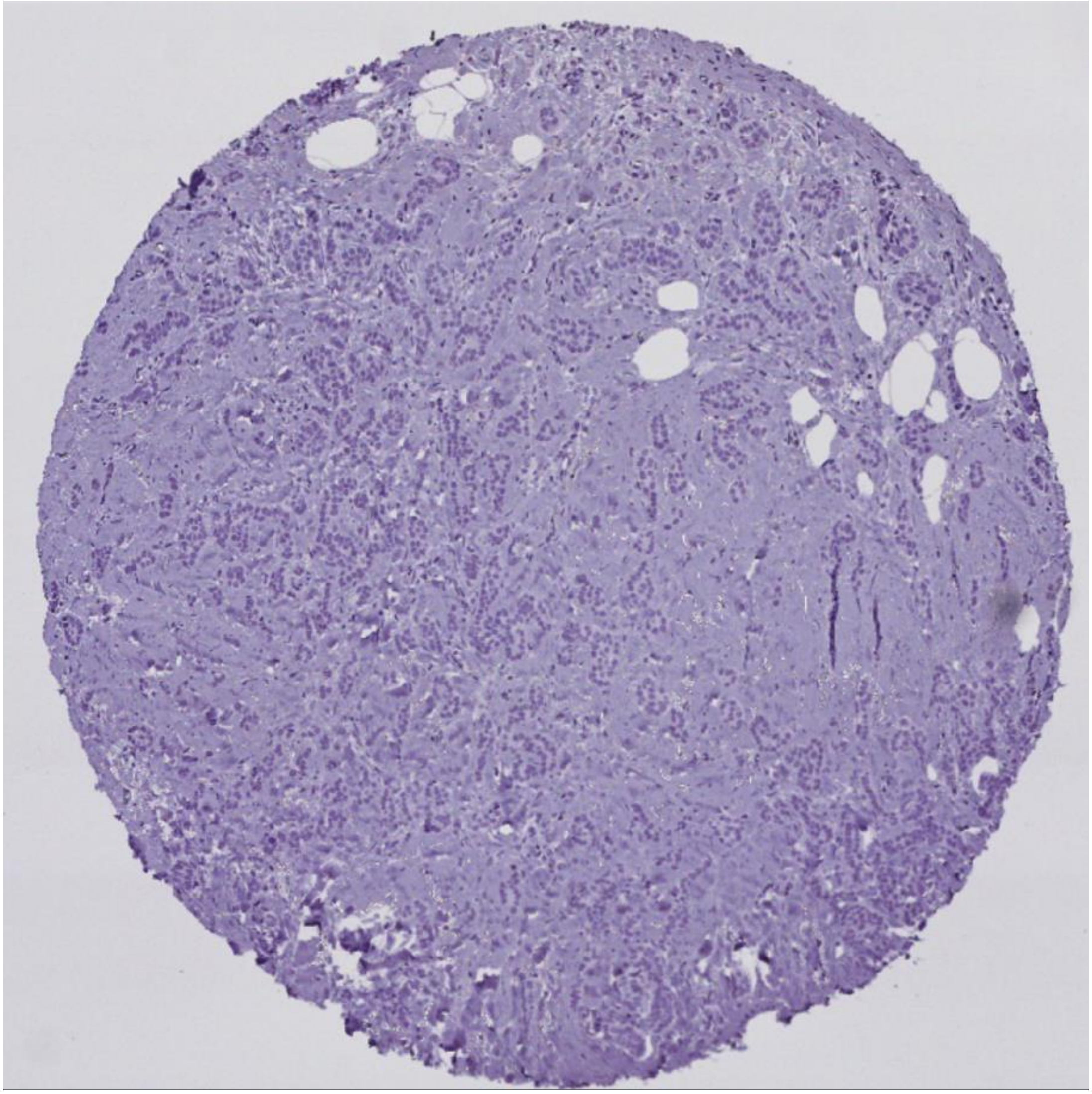

Positive staining (1-9% of lymphocytes)

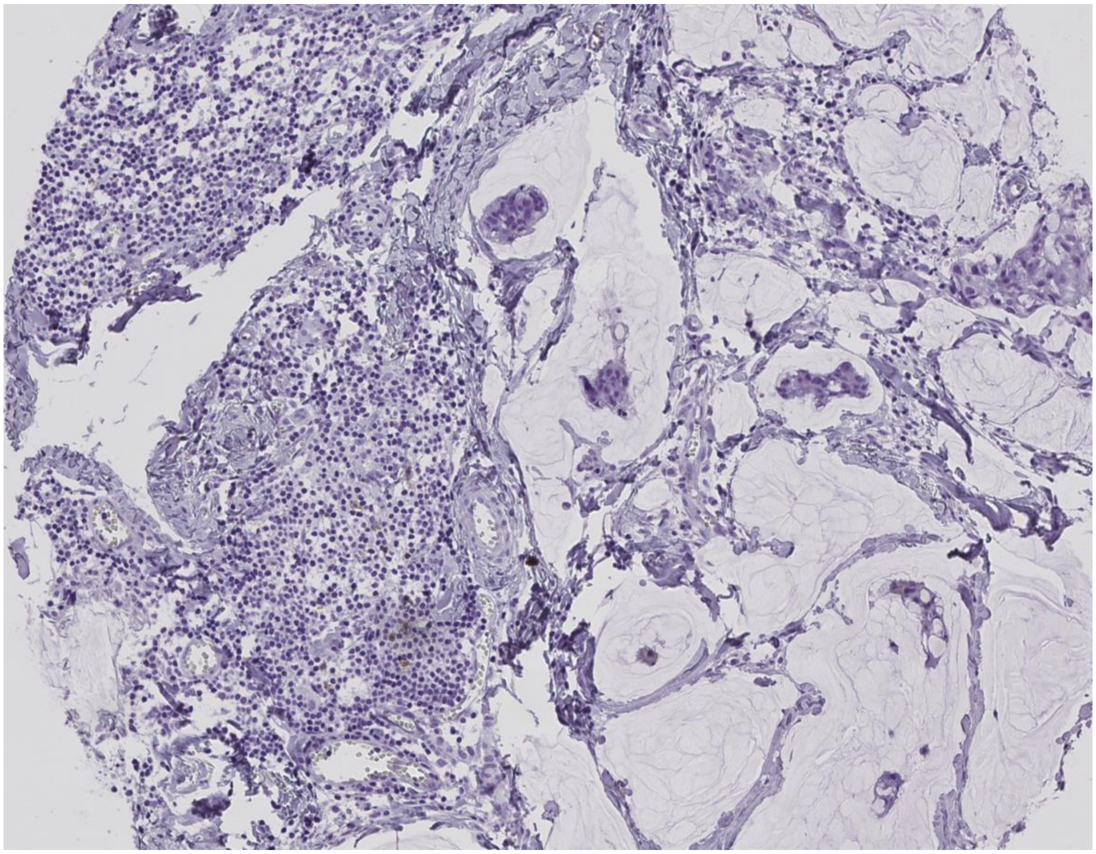

Positive staining (previous image zoomed)

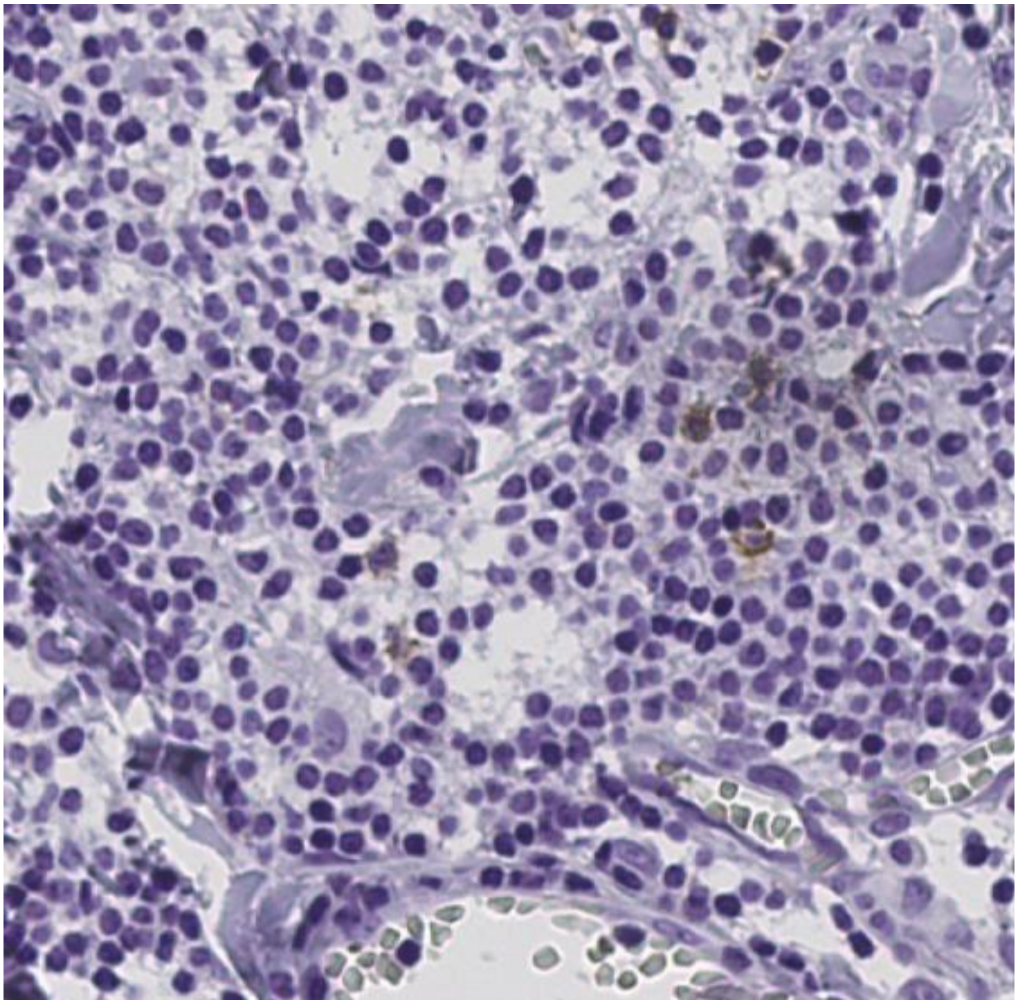

TILs present but no staining

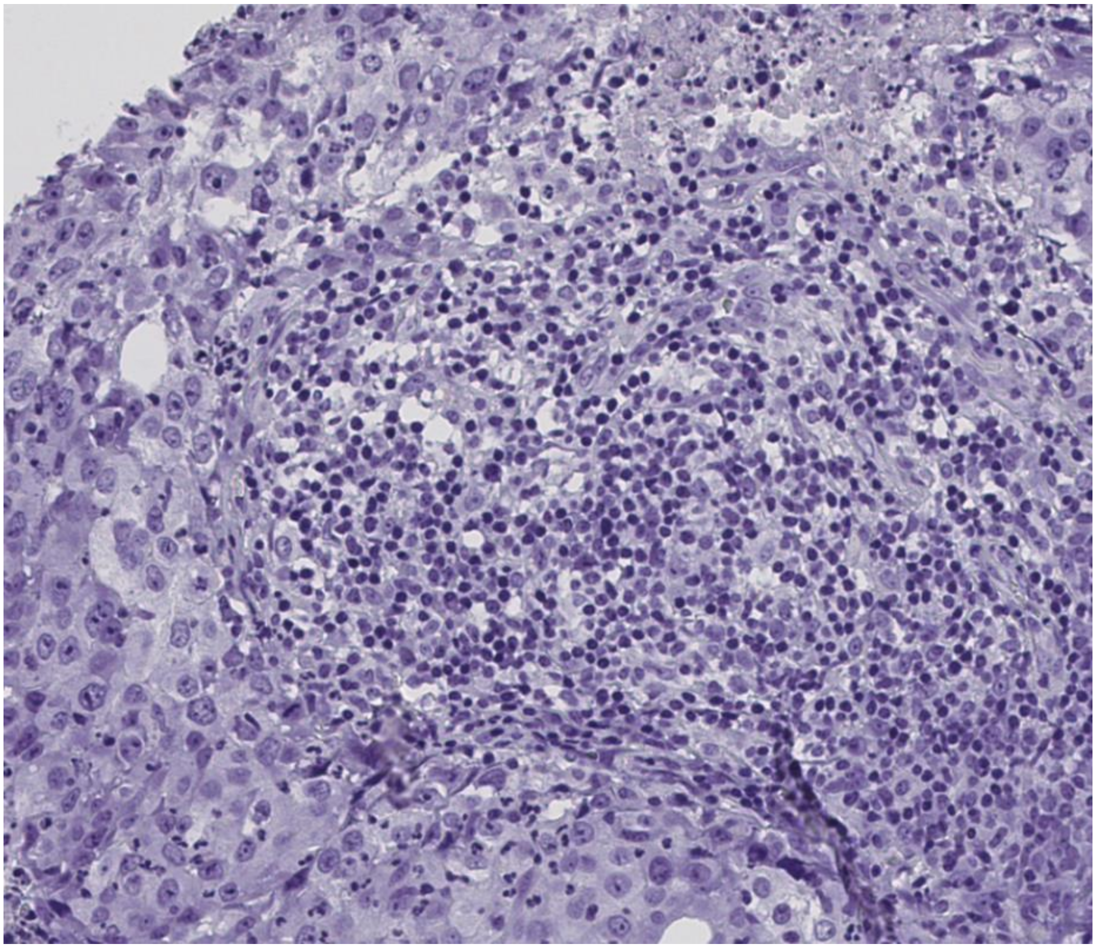

Protocol immunostaining PD-1

Antibody: Cell Marque 315M-95 (NAT105) in a concentration of 1:50.

Tissue block was cut in 4 micrometer sections and then dried in 60°C for 1 hour.

Deparaffinization and pretreatment was performed in pressure cooker with buffer

pH 6.

The following steps were performed in Autostainer *plus*, DAKO staining equipment with Dako kit K8010 solutions, (except for the primary antibody).

Peroxidase Block	5 min.
Primary antibody	30 min.
EnVision (HRP-conjugated polymers)	30 min.
DAB Substrate-chromogen solution	2x5 min.
Hematoxylin (counterstain)	4 min.
Dehydrate and coverslip	

Between every step, rinse was performed with wash buffer.

Protocol immunostaining PD-L1

Instrument: Benchmark Ultra from Ventana

Antibody: PD-L1 (SP142) (RTU) ref.no 740-4859

Pretreatment buffert: Ultra Cell Conditioning (CC1)/43min in 100 degrees C

Detetion kit: OptiView DAB IHC Detetion kit, ref no 760-700

OptiView Amplification Kit, ref no 860-099

Pretreatment buffert: ULTRA Cell Conditioning 1/CC1) 48min/100 degrees C

Antibody incubation: 16 min/37 degrees C

Background staining: Hematoxylin II/8min, ref no. 740-4859
