# Supplementary material for: Combining histological grade, TILs, and the PD-1/PD-L1 pathway to identify immunogenic tumors and de-escalate radiotherapy in early breast cancer: a secondary analysis of a randomized clinical trial
Source: J Immunother Cancer. 2023 May 19;11(5):e006618. doi: 10.1136/jitc-2022-006618 (PMC10201214; doi:10.1136/jitc-2022-006618)
Supplement: Supplementary data [file jitc-2022-006618supp002.pdf]

Using established clinical variables to identify immune responsive tumors in a low-risk cohort

Aims

Immunological biomarkers likely constitute an underutilized source of information in clinical decision making for breast cancer[1]. Despite the large focus on ER-negative subtypes in TILs research, most breast cancers with TILs are ER-positive since the majority of breast tumors are ER-positive[1]. We currently have an incomplete understanding of the role of the immune system in ER-positive breast cancer. We have previously seen that TILs may potentially be used to guide RT individualization in low-risk breast cancer populations. However, to fully be able to utilize this biomarker requires a better understanding of tumor-intrinsic factors affecting immune responsiveness across subtypes rather than within ER-negative subtypes. Furthermore, a better understanding may allow TILs to be used in clinical practice to, in part, guide RT decisions.

We have a unique opportunity of answering the above-mentioned questions in the SweBCG91RT cohort. Our aim is to study if the implications of an immune infiltrate can be predicted by tumor aggressivity, primarily in the form of histological grade, in our low-risk cohort. This may allow for the identification of a subgroup of ER-positive tumors who are immunogenic and may potentially benefit from immunotherapy. Furthermore, we want to investigate if such an understanding may be used to improve RT individualization- an area where additional research is requested by experts within the field[2].

Methods

Study population

The SweBCG91RT cohort. All patients with information on TILs, PD-1, PD-L1, and histological grade will be included. In addition, all patients with high TILs and at least one assessment of PD-1 or PD-L1 of >=1% (even if >=1 TMAs could not be evaluated) as additional TMA evaluations would not change the classification (additional detail for classification under “Analyses”). Among grade II tumors, only those with available gene expression data will be included as gene expression measurements will be used to assess if they will be classified as low- or high-risk (additional details for classification under “Analyses”).

IHC evaluation

Evaluations of PD-1 and PD-L1 from TMAs and TILs from whole sections will be used. Two board-certified pathologists will be evaluating the stainings. The antibodies used for PD-1 and PD-L1, respectively, will be the Cell Marque 315M-95 (NAT105) and Ventana SP142 antibodies. Two cores per

patient and marker are included, and the highest value will be chosen due to the risk of underestimating the degree of positive staining when using TMAs[3]. The same cut-off for positive staining as that in clinical practice will be used (>= 1%) [4].

The reason for including PD-1 and PD-L1 is that they add independent information to TILs which we hypothesize enhances the chances of correctly classifying an immune infiltrate as activated (i.e., having tumor-specific lymphocytes) [1].

Tumor-intrinsic risk group

In a previously unpublished study, we found that a gene expression signature, called Proliferative Index, correlated strongly with histological grade and could be used in a low-risk cohort to predict the biological implications of an immune infiltrate. We, therefore, plan to use histological grade to define immune responsiveness. Grade III tumors will be classified as high-risk and are predicted to benefit from an activated immune infiltrate. Grade I tumors will be classified as low-risk and are not predicted to benefit from an activated immune infiltrate. Grade II tumors constitute a gray zone. They resemble grade I tumors most in terms of Proliferative Index in preliminary assessments and will, therefore, be classified as low-risk unless they have a Proliferative Index above the median of grade III tumors. We believe that classifying the majority of grade II tumors as low-risk in a cohort dominated by ER-positive tumors conforms with the prior literature where an absent or unfavorable prognostic effect from an immune infiltrate is observed[5-7]. The majority of tumors should therefore fall into the low-risk category that does not benefit from an immune infiltrate. Since most tumors are classified as grade II in the SweBCG91RT cohort, this should mean that the majority of grade II tumors would fall into this category. Only grade II tumors with an exceptionally high Proliferative Index (>= median of grade III tumors) will, therefore, be upgraded to high-risk tumors.

Statistics

1. Endpoint: Ipsilateral breast tumor recurrence (IBTR) within 10 years
2. Cox regression analysis to calculate the biologic effect from immune activity and tumor-intrinsic risk group depending on RT in the presence of competing risks
3. Figures: Cumulative incidence functions based on the method described by Fine and Gray with subhazard estimates
4. All analyses are performed in univariable and multivariable analysis including the covariates age, tumor size, ER status, RT
5. Schoenfeld residuals will be used to check proportional hazards assumption

Analyses

6. Classify immune infiltrate into activated or not activated (*immune activity*)
   a. Activated: TILs >= 10% + (PD-1 or PD-L1 >=1%)
   b. Not activated: The remainder of tumors
7. Classify tumors into high- or low-risk (*tumor-intrinsic risk group*)
   a. High-risk: Histological grade III or histological grade II + Proliferative Index >= $\text{median}_{\text{histological grade III}}$
   b. Low-risk: The remainder of tumors (histological grade I or histological grade II + Proliferative Index < $\text{median}_{\text{histological grade III}}$)
8. Analysis of tumor-intrinsic risk group as predictive of immune responsiveness
   a. Interaction test between *tumor-intrinsic risk group* x *immune activity*
9. Analysis of benefit from *immune activity* and *RT* stratified by *tumor-intrinsic risk group*
   a. Cox regression of RT benefit within the four combinations of *immune activity* x *tumor-intrinsic risk group*
   b. Figures cumulative incidence based on Fine and Gray method

1. El Bairi, K., et al., *The tale of TILs in breast cancer: A report from The International Immuno-Oncology Biomarker Working Group.* NPJ Breast Cancer, 2021. **7**(1): p. 150.
2. Kaidar-Person, O., P. Poortmans, and R. Salgado, *Genomic-adjusted radiation dose to personalise radiotherapy.* Lancet Oncol, 2021. **22**(9): p. 1200-1201.
3. Sobral-Leite, M., et al., *Assessment of PD-L1 expression across breast cancer molecular subtypes, in relation to mutation rate, BRCA1-like status, tumor-infiltrating immune cells and survival.* Oncoimmunology, 2018. **7**(12): p. e1509820.
4. Schmid, P., et al., *Atezolizumab and Nab-Paclitaxel in Advanced Triple-Negative Breast Cancer.* N Engl J Med, 2018. **379**(22): p. 2108-2121.
5. Sobral-Leite, M., et al., *Cancer-immune interactions in ER-positive breast cancers: PI3K pathway alterations and tumor-infiltrating lymphocytes.* Breast Cancer Res, 2019. **21**(1): p. 90.
6. Liu, S., et al., *Prognostic significance of FOXP3+ tumor-infiltrating lymphocytes in breast cancer depends on estrogen receptor and human epidermal growth factor receptor-2 expression status and concurrent cytotoxic T-cell infiltration.* Breast Cancer Res, 2014. **16**(5): p. 432.
7. Johansson, A., et al., *Clinical and molecular characteristics of estrogen receptor-positive ultralow risk breast cancer tumors identified by the 70-gene signature.* Int J Cancer, 2022.
